# The Physical Characteristics of Elite Female Rugby Union Players

**DOI:** 10.3390/ijerph17186457

**Published:** 2020-09-04

**Authors:** Logan Posthumus, Campbell Macgregor, Paul Winwood, Jamie Tout, Lillian Morton, Matthew Driller, Nicholas Gill

**Affiliations:** 1 Faculty of Health, Education and Environment, Toi Ohomai Institute of Technology, Tauranga 3112, New Zealand; campbell.macgregor@toiohomai.ac.nz (C.M.); paul.winwood@toiohomai.ac.nz (P.W.); 2 Te Huataki Waiora School of Health, The University of Waikato, Hamilton 3216, New Zealand; nicholas.gill@nzrugby.co.nz; 3 New Zealand Rugby, Wellington 6011, New Zealand; jt@xlr8.co.nz (J.T.); lillianmortonnutrition@gmail.com (L.M.); 4 School of Applied and Social Sciences, Central Queensland University, Rockhampton 4701, Australia; 5 School of Sport and Recreation, Auckland University of Technology, Auckland 0627, New Zealand; 6 School of Allied Health, Human Services and Sport, La Trobe University, Melbourne 3086, Australia; m.driller@latrobe.edu.au

**Keywords:** bone mineral density, lean mass, fat mass, fat percentage, skinfolds

## Abstract

This study explored the anthropometric and body composition characteristics of elite female rugby union players, comparing between and within different playing positions. Thirty elite female rugby union players (25.6 ± 4.3 y, 171.3 ± 7.7 cm, 83.5 ± 13.9 kg) from New Zealand participated in this study. Physical characteristics were assessed using anthropometric (height, body mass, skinfolds) and body composition (dual-energy X-ray absorptiometry) measures. Forwards were significantly taller (*p* < 0.01; *d* = 1.34), heavier (*p* < 0.01; *d* = 2.19), and possessed greater skinfolds (*p* < 0.01; *d* = 1.02) than backs. Forwards also possessed significantly greater total (*p* < 0.01; *d* = 1.83–2.25) and regional (*p* < 0.01; *d* = 1.50–2.50) body composition measures compared to backs. Healthy bone mineral density values were observed in both forwards and backs, with significantly greater values observed at the arm (*p* < 0.01; *d* = 0.92) and femoral neck (*p* = 0.04; *d* = 0.77) sites for forwards. Tight-five players were significantly heavier (*p* = 0.02; *d* = 1.41) and possessed significantly greater skinfolds (*p* < 0.01; *d* = 0.97) than loose-forwards. Tight-five also possessed significantly greater total body composition measures (*p* < 0.05; *d* = 0.97–1.77) and significantly greater trunk lean mass (*p* = 0.04; *d* = 1.14), trunk fat mass (*p* < 0.01; *d* = 1.84), and arm fat mass (*p* = 0.02; *d* = 1.35) compared to loose-forwards. Specific programming and monitoring for forwards and backs, particularly within forward positional groups, appear important due to such physical characteristic differences.

## 1. Introduction

Rugby union (RU) is a high contact field-based team sport which is played all over the world at junior, senior, sub-elite, and elite levels by both males and females. In recent years, RU has become increasingly popular with more elite competitions and matches being contested by female RU players each year [1]. The same rules apply for both sexes, in which a game is contested over two 40 min halves by two teams consisting of 15 players aside comprised of eight forwards (numbers 1–8) and seven backs (numbers 9–15), with eight reserves on the bench (side-line). The game is intermittent in nature which is characterized by repeated bouts of high-intensity exercise (running, sprinting, tackling, scrummaging, rucking, and mauling) interspersed with periods of lower intensity exercise (standing, walking, and jogging). Both provincial [2] and elite [3] female RU players have been shown to cover approximately 5500 to 6400 m during a game, with elite female players demonstrating an average heart rate ~161 bpm and ~700 impacts during a game [3].

Specific anthropometric (height, body mass, skinfold) and body composition (lean mass, fat mass, bone mass) characteristics are required for specific positions in order to meet the physical demands of the sport [4]. Specifically, adequate amounts of body mass in the form of lean mass, fat mass, and bone mass appear to be crucial in order to withstand the frequency and intensity of collisions during offensive and defensive match-play [5]. Meanwhile, an optimal body composition that promotes lean mass and reduces fat mass are also desired to support the development of power, speed, and aerobic fitness, which are important factors to repeatedly perform and complete tasks at a high level [6].

Despite the known positional differences regarding anthropometrics and body composition within elite male RU players [5,7,8,9,10,11,12], body composition research within elite female RU players is limited. Elite female RU forwards have been shown to be significantly taller, heavier, and possess greater skinfolds and predicted fat percentage compared to backs [13,14,15]. However, a more recent study [16] demonstrated that forwards were larger and possessed more lean and fat mass than backs, but no significant differences were reported. Further research is required to understand the positional differences within these athletes, as it is well understood that specific roles between and within forwards and backs vary greatly due to the demands of the sport [4,17].

Body composition within elite female RU players has been predominantly determined using skinfolds and predictive equations to provide an estimated body fat percentage [13,14,15]. More advanced methods have also been implemented to assess body composition within these athletes, such as air displacement (BodPod) [16]. However, a method that has gained popularity for body composition analysis within elite male RU players is the use of dual-energy X-ray absorptiometry (DXA) [5,7,8,9,10,11,12,18]. Implementing DXA to assess body composition allows the measurement of lean, fat, and bone mass for both total and regional body compartments [19]. Moreover, DXA is the gold standard for measuring bone mineral content (BMC) and bone mineral density (BMD), which provide valuable information regarding the bone health of an athlete [20], which is particularly important among elite female athletes due to associations with female athlete triad [21].

Although a recent study has investigated the body composition of elite female rugby league (RL) players using DXA [22], no study has investigated the body composition and BMD of elite female RU players using DXA. Due to the differences in match-play demands observed between RU and RL [23,24], and between female and male RU [3,25], specific body composition information is required for elite female RU players. This information would be beneficial for talent identification purposes, and for practitioners developing female RU players by providing body composition benchmarks to inform training and nutrition programs. Therefore, the purpose of this study was to investigate the anthropometric and body composition characteristics of New Zealand (NZ) elite female RU players. Comparisons between forwards and backs, including differences within both forward and back positional groups, were explored. It was hypothesized that forwards would possess significantly greater anthropometric, total, and regional body composition measures compared to backs, and significant differences would be observed among forward and back positional groups.

## 2. Materials and Methods

### 2.1. Participants and Study Design

Thirty elite female RU players (25.6 ± 4.3 y, 171.3 ± 7.7 cm, 83.5 ± 13.9 kg) from NZ were recruited for this study. Players were first categorized into forwards (*n* = 15) and backs (*n* = 15) for analysis and comparison, followed by positional groups within forwards (tight-five (TF) and loose-forwards (LF)) and backs (inside-backs (IB) and outside-backs (OB)). The TF (*n* = 9) were comprised of hookers (*n* = 3), props (*n* = 4), and locks (*n* = 2), while LF (*n* = 6) were comprised of flankers and no. 8’s. The IB (*n* = 8) were comprised of half-backs (*n* = 2), first-fives (*n* = 3), and second-fives (*n* = 3), while OB (*n* = 7) were comprised of centers (*n* = 2), wingers, and full backs (*n* = 5). All testing took place during the middle of the in-season period and all participants provided informed consent. The research was approved by the University of Waikato Human Research Ethics Committee (HREC 2019#02).

### 2.2. Anthropometrics

All anthropometric measures were collected using methods previously described [12]. Body mass was assessed using electronic scales (SECA, Birmingham, UK) to 0.1 kg accuracy upon waking with bladder voided. Height was then immediately assessed using a stadiometer (SECA, Birmingham, UK) to 0.5 cm accuracy. A Level 3 International Society for the Advancement of Kinanthropometry (ISAK) accredited anthropometrist with a technical error of measurement of 1.8% carried out sum of eight site skinfold measurements on all players. Skinfolds were taken using Harpenden callipers (British Indicators, Hertfordshire, UK) to 0.1 mm accuracy. Sum of eight site skinfold measurements from the following sites; biceps, triceps, subscapular, abdominal, supraspinale, iliac crest, mid-thigh and medial calf were made on the right side of the body using ISAK techniques previously described by Norton and Colleagues [26]. All anthropometric equipment was calibrated as recommended by the manufacturer’s guidelines.

### 2.3. Body Composition

Using previously described methods [12], total and regional body composition (lean mass, fat mass, fat percentage, BMC, and BMD), were measured with a fan-beam DXA scanner (Hologic Discovery A, Hologic, Bedford, MA), with analyses performed using Apex 4.5.2 software (Hologic, Bedford, MA) using a whole-body scan protocol. The following clinical site measures for BMD; anterior-posterior lumbar spine (L2–L4) and left hip (femoral neck, trochanter, intertrochanter, and total hip) were included. All scanning procedures were standardized for all participants following the guidelines of the DXA manufacturer and the standards outlined by the International Society for Clinical Densitometry (ISCD). The scanner was tested for consistent calibration daily as per manufacturer guidelines for quality control purposes.

The quality control, acquisition, analysis, and interpretation of all scans were performed by the same experienced and Certified Clinical Densitometrist (CCD), therefore maintaining consistency and reliability of the scan results [27]. The head was included in the analysis for all total body measures and all scans were undertaken using the array mode. Scanned data was separated into axial, appendicular, and whole-body bone values. In the Bone Density Research Laboratory site, coefficients of variation (CV) for precision and accuracy for the spine phantom were 0.3% and 0.4%, respectively. The in vivo precision CV for the CCD technician are 0.9% for total body, 0.6% for lumbar spine (L2–L4), and 0.2% for left total hip BMD sites. The prevalence of osteoporosis and osteopenia were estimated based on the World Health Organization (WHO) classifications (t-score > −1.0 = normal; t-score −1.1 to −2.4 = osteopenia; t-score < −2.5 = osteoporosis) using the young adult reference database.

All participants were scanned on the same day within a five-hour window (1000–1500 h), with all participants consuming breakfast and fluids (~500 g) at the same time (0830 h). Participants were required to remove all metal items from their body and were scanned wearing tight-fitting sport shorts and top that were free from zips, studs, and/or metal objects. Participants were then instructed to lay supine on the scanning bed as still as possible for the duration of the scan. All protocols followed previously described techniques to maximize technical reliability and minimize error [19,28], however, players were unable to be scanned in a fasted state.

### 2.4. Statistical Methods

All data expressed as means and standard deviation (SD). A 95% confidence interval (95% CI) was presented alongside mean difference and standard error (SE) for all variables. Tests of normality (Shapiro–Wilk) were carried out, which revealed normally distributed data. Differences between forwards and backs and within positional groups (TF vs LF and IB vs OB) were compared using an independent *t*-test. Statistical significance was set as *p* < 0.05 for analyses. Henceforth, when the term ‘significant’ and ‘significantly’ is stated, it denotes a statistically significant difference. Effect sizes were calculated using the Cohen’s *d* method with the following thresholds: *d* = *small* 0.20–0.49, *medium* 0.50–0.79, and *large* > 0.80 [29]. All statistical analyses were conducted using SPSS v24 for Windows (IBM, New York, NY, USA).

## 3. Results

### 3.1. Analysis between Forwards and Backs

The demographics, anthropometrics, and total body composition for all players and by position can be observed in Table 1. Forwards were significantly taller (*p* < 0.01; *d* = 1.34), heavier (*p* < 0.01; *d* = 2.19), and had significantly greater skinfold totals (*p* < 0.01; *d* = 1.02) than backs. Forwards possessed significantly greater total lean mass (*p* < 0.01; *d* = 1.83), fat mass (*p* < 0.01; *d* = 2.25), fat percentage (*p* < 0.01; *d* = 1.87), and BMC (*p* < 0.01; *d* = 1.40) than backs.

Regional body composition for all players and by position can be observed in Table 2. Forwards possessed significantly greater measures across the trunk, arms, and legs for lean mass (*p* < 0.01; *d* = 1.77, 1.53, 1.50, respectively), fat mass (*p* < 0.01; *d* = 1.75, 1.87, 2.50, respectively), and BMC (*p* < 0.01; *d* = 0.88, 1.51, 1.48, respectively) compared to backs. No clear differences were observed for age between forwards and backs.

### 3.2. BMD between Forwards and Backs

The BMD for all players and by position can be observed in Table 3. No significant differences were observed between forwards and backs for total body (*p* = 0.63, *d* = 0.33) and total hip (*p* = 0.73, *d* = 0.50) BMD. However, forwards demonstrated significantly greater BMD in the arms (*p* < 0.01, *d* = 0.92) and femoral neck (*p* = 0.04; *d* = 0.77) compared to backs. There were no significant differences between forwards and backs for total body and clinical site BMD t-scores.

### 3.3. Analysis within Forward and Back Positional Groups

Within position differences for forwards and backs can be observed in Table 4. The TF possessed significantly greater body mass (*p* = 0.02; *d* = 1.41) and skinfolds (*p* < 0.01; *d* = 2.01) compared to LF. The TF demonstrated significantly greater total lean mass (*p* = 0.04; *d* = 0.97), fat mass (*p* < 0.01; *d* = 1.75), and fat percentage (*p* < 0.01; *d* = 1.77) than LF. Regarding regional body composition, TF presented significantly greater trunk lean mass (*p* = 0.04; *d* = 1.14), trunk fat mass (*p* < 0.01; *d* = 1.84), and arm fat mass (*p* = 0.02; *d* = 1.35) compared to LF. No significant differences were observed between IB and OB for all measures.

## 4. Discussion

The purpose of this study was to investigate the anthropometric and body composition characteristics of NZ elite female RU players. Comparisons were made between forward and back positions, including analysis of within position differences. As hypothesized, forwards demonstrated significantly greater values across all anthropometric, total, and regional body composition measures than backs. Additionally, forwards presented greater BMD across all sites with significant differences present at the arms and femoral-neck compared to backs. Interestingly, only forward positional groups demonstrated significant differences, in which TF possessed significantly greater anthropometric, total, and regional body composition measures compared to LF. This study is the first to assess the body composition characteristics of elite female RU players using DXA, and the first to explore differences between forward and back positional groups.

The anthropometric and body composition differences between forwards and backs are consistent with previous research within both elite female [13,14,15,16] and elite male RU players [5,7,8,9,10,11,12]. Irrespective of sex, forwards require these specific physical characteristics to meet match demands where being taller is desirable in order to gain and retain possession of the ball during line outs, while greater body mass may assist in gaining and retaining ball possession during scrums, rucks, and mauls [4]. Meanwhile, backs are generally shorter, lighter, and leaner in order to achieve higher speeds in more open spaces in an attempt to outmaneuver opponents to create scoring opportunities [4]. Studies within male RU players have also demonstrated that as playing level increases, players are generally heavier with greater lean mass, whilst possessing lower skinfold and body fat percentage [17]. Researchers have also suggested that being taller and heavier appear to be important key performance indicators for World Cup success in elite male RU players [30,31]. More research is required to determine whether such trends exist within elite female RU players.

Within the current study, both forwards and backs were taller and heavier than elite female RU players reported in previous studies [13,14,15,16]. Although forwards and backs within the current study were substantially heavier compared to other elite female RU players [13,14,15,16], the current players possessed noticeably lower skinfolds, even when comparing sum of eight-site skinfolds within this study, with sum of seven-site skinfolds within previous studies [14,15]. The measured fat percentage using DXA for forwards and backs within this study were considerably lower than the predicted fat percentage reported for elite South African players [14] but were similar to elite English players [13]. Additionally, forwards within the current study demonstrated greater total body lean and fat mass, but lower fat percentage compared to elite Scottish female forwards. Meanwhile, backs demonstrated greater total body lean mass and considerably less fat mass and fat percentage compared to elite Scottish female RU backs [16].

It is important to note that considerably less body mass was reported within the previous studies [14,16], but particularly within English players [13] compared to the current study. These comparisons highlight the substantial increases in size and in particular body mass within elite female RU players as the sport has progressed throughout the years [13,14,15,16]. Moreover, differences in size and body mass may also be attributed to cultural and ethnicity differences between countries [5,7,8,18]. Increases in body mass, but particularly lean mass, are desired due to the contractile element of muscle mass aiding force production, thus positively influencing power to weight ratio and the expression of strength, power, speed and momentum [7]. These are all important factors for attacking and defending within RU and may be further enhanced by limiting/reducing the amount of non-functional fat mass [12]. Too much fat mass may also negatively influence energy expenditure, thermoregulation, and the ability to repeatedly perform tasks within RU match-play [6]. This information supports the need for more current studies reporting the physical and fitness profiles of elite female RU players.

Until now, no research has been available regarding the regional body composition of elite female RU players. The results of this study align with previous research within elite male RU players which demonstrated significantly greater amounts of regional lean mass, fat mass, and BMC in forwards compared to backs [5,7]. When compared to elite female RL players, elite female RU forwards possess greater lean mass at all regional sites, while possessing similar amounts of fat mass at the arms and legs, but less fat mass at the trunk compared to elite female RL forwards [22]. Meanwhile, elite female RU backs demonstrated greater lean mass while possessing lower amounts of fat mass at all regional sites compared to elite female RL backs [22].

When comparing the positional differences within forwards and backs, only forward positional groups demonstrated significant differences. These findings suggest that positional groups within backs are more homogeneous than within forward positional groups. Therefore, more individualized training and nutrition programs may be required for forwards compared to backs. Moreover, TF forwards could potentially aim to decrease fat mass, particularly in the trunk and arms to improve power to weight ratio and acceleration capability [7]. However, further research is required to understand whether or not this extra fat mass demonstrated by TF players is important for the demands of the position. A general consensus is that greater fat mass within collision-based athletes may be required to better deal with impacts due to the “cushioning” effect of fat tissue [22]. Therefore, determining how much fat mass is optimal within female RU players would be valuable.

Interestingly, when comparing the percentage difference for all measures between forwards and backs within the current study to NZ elite male RU players [12], similar differences can be observed. The percentage difference for anthropometric and total body composition measures between forwards and backs within elite females were very similar to elite males [12]. This information suggests that regardless of sex, RU may produce similar positional differences in relation to anthropometrics and body composition. However, it is important to note that females possess unique physiological differences compared to males such as, constant changes in the female sex hormone milieu throughout the menstrual cycle and therefore, across the training program [32]. Due to the effect’s hormones have on the body, training, and adaptation, these areas within female athletes need to be carefully considered when developing training and nutrition plans [32].

Future research could assess the physical characteristics derived from DXA alongside fitness measures in order to examine the associations between body composition and physical performance within elite female RU players. Furthermore, exploring the body composition changes using DXA and changes in fitness measures during pre-season and in-season phases would provide valuable information regarding seasonal changes in physical and fitness characteristics. Carrying out DXA scans to track longitudinal changes in body composition to inform training and nutrition programs also allows for the analysis of BMD. Although low BMD does not appear to be a concern within this group of elite athletes, DXA is valuable to identify any athletes with low BMD. Greater insight into the movement demands, training load, and energy requirements of elite female RU players would also be valuable due to the associations between training and nutrition for optimizing body composition [33,34].

A limitation within the current study was the relatively small sample size of elite players, particularly when exploring the within position differences for forwards and backs. A larger sample size would have provided a greater representation of the within position differences and would have allowed further exploration into forward (front row, second row, back row) and back (inside-back, midfield, outside-back) positions. However, this sample size should be appropriate to provide valuable information for talent identification, coaching, and sport science staff. Another limitation within this study was the fact that participants could not be in a fasted state before DXA scans. Researchers should strive to carry out DXA protocols in a fasted state, however within elite sporting environments this can be a challenging task. Although not optimal, all participants consumed less than 500 g of food and fluids which has been shown to maintain low biological measurement error when using DXA [35].

## 5. Conclusions

In conclusion, forwards were significantly larger than backs, possessing greater stature, body mass, skinfolds, and total and regional body composition measures. Total and regional BMD values are within normal ranges, with forwards demonstrating significantly greater arm and femoral neck BMD. This study presented novel findings regarding the significant differences between forward positional groups, in which TF were significantly larger than LF. However, no significant differences were observed between back positional groups. This information may be useful for coaches and sport science staff when developing training and nutrition programs for female RU players. It may also provide useful benchmarks that can assist talent identification staff and the development of future female RU players.

## Figures and Tables

**Table 1:** Demographics, anthropometrics and total body composition characteristics of elite female rugby union (RU) players.

	All Players	Position		Mean Diff	95% CI	% Diff; ES
		Forwards	Backs			
	(*n* = 30)	(*n* = 15)	(*n* = 15)	(± SE)		
Age (y)	25.6 ± 4.3	25.3 ± 3.6	25.8 ± 5.0	−0.5 ± 1.6	−3.7, 2.8	−1.8; −0.11
Height (cm)	171.3 ± 7.7	175.6 ± 6.3 *	167.0 ± 6.6	8.6 ± 2.4	3.8, 13.5	5.2; 1.34
Body Mass (kg)	83.5 ± 13.9	93.7 ± 10.9 *	73.3 ± 7.5	20.5 ± 3.4	13.5, 27.5	27.9; 2.19
Sum8SF (mm)	111.3 ± 36.7	128.2 ± 36.6 *	94.4 ± 29.0	33.8 ± 12.1	9.1, 58.5	35.8; 1.02
Lean Mass (kg)	60.9 ± 7.8	66.2 ± 6.3 *	55.6 ± 5.3	10.6 ± 2.1	6.3, 14.9	19.0; 1.83
Fat Mass (kg)	20.3 ± 6.6	25.3 ± 5.4 *	15.4 ± 3.1	9.9 ± 1.6	6.6, 13.2	64.5; 2.25
Fat % (%)	23.6 ± 4.2	26.5 ± 3.1 *	20.8 ± 3.0	5.8 ± 1.1	3.5, 8.1	27.7; 1.87
BMC (kg)	2.9 ± 0.3	3.1 ± 0.3 *	2.7 ± 0.2	0.4 ± 0.1	0.2, 0.5	13.1; 1.40
BMD (g/cm^2^)	1.24 ± 0.08	1.26 ± 0.08	1.23 ± 0.07	0.03 ± 0.03	−0.03, 0.08	2.1; 0.33

Mean ± standard deviation (SD) reported for all players, forwards and backs. Mean Diff (±SE) = mean difference ± standard error of mean difference, 95% CI = 95% confidence interval (lower limit, upper limit), % Diff = percentage difference, ES = effect size, BMC = bone mineral content, BMD = bone mineral density, Sum8SF = sum of eight-site skinfolds. * Statistically significant difference (*p* < 0.05) compared to backs.

**Table 2:** Regional body composition characteristics of elite female RU players.

		All Players	Position		Mean Diff	95% CI	% Diff; ES
			Forwards	Backs			
		(*n* = 30)	(*n* = 15)	(*n* = 15)	(± SE)		
Trunk	Lean Mass (kg)	29.4 ± 4.2	32.2 ± 3.7 *	26.6 ± 2.5	5.6 ± 1.2	3.3, 7.9	21.0; 1.77
	Fat Mass (kg)	8.5 ± 3.5	10.8 ± 3.4 *	6.2 ± 1.6	4.6 ± 1.0	2.6, 6.6	75.3; 1.75
	BMC (kg)	0.90 ± 0.12	0.94 ± 0.12 *	0.85 ± 0.10	0.10 ± 0.04	0.01, 0.18	11.2; 0.88
Arms	Lean Mass (kg)	6.9 ± 0.9	7.5 ± 0.8 *	6.3 ± 0.7	1.1 ± 0.3	0.6, 1.7	17.9; 1.53
	Fat Mass (kg)	2.3 ± 0.9	2.9 ± 0.8 *	1.7 ± 0.4	1.2 ± 0.2	0.7, 1.7	71.7; 1.87
	BMC (kg)	0.40 ± 0.06	0.44 ± 0.05 *	0.37 ± 0.04	0.07 ± 0.02	0.04, 0.11	19.5; 1.51
Legs	Lean Mass (kg)	21.5 ± 3.0	23.3 ± 2.4 *	19.6 ± 2.5	3.7 ± 0.9	1.8, 5.5	18.6; 1.50
	Fat Mass (kg)	8.5 ± 2.6	10.5 ± 1.8 *	6.5 ± 1.4	4.0 ± 0.6	2.8, 5.2	62.2; 2.50
	BMC (kg)	1.08 ± 0.14	1.17 ± 0.13 *	1.00 ± 0.10	0.17 ± 0.04	0.08, 0.26	17.0; 1.48

Mean ± SD reported for all players, forwards and backs. Mean Diff (± SE) = mean difference ± standard error of mean difference, 95% CI = 95% confidence interval (lower limit, upper limit), % Diff = percentage difference, ES = effect size, BMC = bone mineral content. * Statistically significant difference (*p* < 0.05) compared to backs.

**Table 3:** Total and clinical site BMD (g/cm^2^) of elite female RU players.

	All Players	Position		Mean Diff	95% CI	% Diff; ES
		Forwards	Backs			
	(*n* = 30)	(*n* = 15)	(*n* = 15)	(SE)		
Arms	0.84 ± 0.06	0.87 ± 0.07 *	0.82 ± 0.05	0.05 ± 0.02	0.01, 0.10	6.5; 0.92
Legs	1.29 ± 0.09	1.31 ± 0.10	1.27 ± 0.07	0.05 ± 0.03	−0.02, 0.11	3.7; 0.54
Ribs	0.77 ± 0.07	0.78 ± 0.07	0.76 ± 0.06	0.02 ± 0.02	−0.03, 0.07	3.1; 0.36
Spine	1.17 ± 0.13	1.19 ± 0.14	1.15 ± 0.12	0.04 ± 0.05	−0.05, 0.14	3.7; 0.34
Pelvis	1.55 ± 0.18	1.56 ± 0.17	1.55 ± 0.19	0.01 ± 0.07	−0.12, 0.15	0.7; 0.06
Head	2.19 ± 0.24	2.20 ± 0.27	2.18 ± 0.21	0.03 ± 0.09	−0.16, 0.21	1.2; 0.11
Total	1.24 ± 0.08	1.26 ± 0.08	1.23 ± 0.07	0.03 ± 0.03	−0.03, 0.08	2.1; 0.33
Total t-score	1.58 ± 0.97	1.80 ± 1.03	1.31 ± 0.86	0.49 ± 0.39	−0.32, 1.30	37.5; 0.51
Clinical Sites						
Lumbar (L2–L4)	1.31 ± 0.15	1.36 ± 0.14	1.25 ± 0.13	0.10 ± 0.05	0.00, 0.21	8.1; 0.73
Femoral Neck	1.12 ± 0.11	1.16 ± 0.10 *	1.07 ± 0.12	0.08 ± 0.04	0.00, 0.16	7.8; 0.77
Trochanter	0.94 ± 0.10	0.95 ± 0.10	0.94 ± 0.11	0.01 ± 0.04	−0.07, 0.09	0.8; 0.07
Intertrochanter	1.39 ± 0.14	1.42 ± 0.13	1.35 ± 0.14	0.07 ± 0.05	−0.03, 0.17	5.3; 0.53
Total hip	1.21 ± 0.11	1.24 ± 0.10	1.18 ± 0.12	0.06 ± 0.04	−0.03, 0.14	4.7; 0.50
Clinical site t-score	1.99 ± 1.46	2.46 ± 1.41	1.44 ± 1.37	1.03 ± 0.57	−0.16, 2.21	71.4; 0.74

Mean ± SD reported for all players, forwards and backs. Mean Diff (±SE) = mean difference ± standard error of mean difference, 95% CI = 95% confidence interval (lower limit, upper limit), % Diff = percentage difference, ES = effect size. * Statistically significant difference (*p* < 0.05) compared to backs.

**Table 4:** Demographics, anthropometrics, total and regional body composition within forward and back positional groups.

	Forward Positional Groups (*n* = 15)					Back Positional Groups (*n* = 15)				
	TF	LF	Mean Diff			IB	OB	Mean Diff		
	(*n* = 9)	(*n* = 6)	(± SE)	95% CI	% Diff; ES	(*n* = 8)	(*n* = 7)	(± SE)	95% CI	% Diff; ES
Age (y)	25.2 ± 3.0	25.5 ± 4.8	−0.3 ± 2.0	−4.6, 4.0	−1.1; −0.07	26.8 ± 4.9	24.7 ± 5.3	2.0 ± 2.6	−3.6, 7.7	8.2; 0.40
Anthropometrics										
Height (cm)	174.8 ± 7.6	176.8 ± 3.9	−2.1 ± 3.4	−9.4, 5.3	−1.2; −0.32	168.8 ± 8.0	164.9 ± 4.3	3.8 ± 3.4	−3.5, 11.2	2.3; 0.58
Weight (kg)	98.8 ± 10.9 *	86.0 ± 4.9	12.8 ± 4.8	2.5, 23.1	14.9; 1.41	73.6 ± 5.8	72.9 ± 9.6	0.7 ± 4.0	−8.0, 9.4	0.9; 0.09
Sum8SF (mm)	149.2 ± 28.8 *	96.8 ± 20.9	52.4 ± 13.7	22.8, 82.1	54.2; 2.01	95.5 ± 31.2	93.2 ± 28.9	2.3 ± 15.6	−31.4, 36.0	2.5; 0.08
Body Composition										
Total Lean Mass (kg)	68.5 ± 7.1 *	62.9 ± 2.3	5.6 ± 3.1	−1.0, 12.2	8.9; 0.97	55.5 ± 4.9	55.8 ± 6.0	−0.3 ± 2.8	−6.4, 5.8	−0.5; −0.05
Total Fat Mass (kg)	28.2 ± 4.7 *	21.0 ± 3.2	7.3 ± 2.2	2.5, 12.0	34.6; 1.75	15.8 ± 2.1	14.9 ± 4.0	0.9 ± 1.6	−2.5, 4.5	6.6; 0.31
Total Fat % (%)	28.2 ± 2.4 *	24.0 ± 2.3	4.2 ± 1.3	1.5, 6.9	17.5; 1.77	21.4 ± 2.4	20.1 ± 3.7	1.3 ± 1.6	−2.1, 4.8	6.6; 0.43
Trunk Lean Mass (kg)	33.7 ± 3.7 *	29.9 ± 2.6	3.8 ± 1.8	0.2, 7.6	12.7; 1.14	27.2 ± 2.2	25.9 ± 2.7	1.3 ± 1.3	−1.4, 4.1	5.1; 0.54
Trunk Fat Mass (kg)	12.7 ± 2.9 *	8.0 ± 1.9	4.7 ± 1.3	1.8, 7.6	58.6; 1.84	6.2 ± 1.3	6.1 ± 2.0	0.1 ± 0.9	−1.8, 1.9	0.4; 0.02
Arm Lean Mass (kg)	7.7 ± 0.8	7.2 ± 0.6	0.5 ± 0.4	−0.4, 1.3	6.7; 0.65	6.2 ± 0.6	6.5 ± 0.9	−0.3 ± 0.4	−1.1, 0.6	−3.8; −0.33
Arm Fat Mass (kg)	3.2 ± 0.8 *	2.3 ± 0.6	0.9 ± 0.4	0.2, 1.8	41.5; 1.35	1.8 ± 0.2	1.5 ± 0.4	0.3 ± 0.2	−0.1, 0.7	19.0; 0.86
Leg Lean Mass (kg)	23.8 ± 2.8	22.6 ± 1.5	1.2 ± 1.3	−1.5, 3.9	5.2; 0.50	19.1 ± 2.3	20.3 ± 2.8	−1.2 ± 1.3	−4.0, 1.7	−5.7; −0.46
Leg Fat Mass (kg)	11.1 ± 1.5	9.5 ± 1.9	1.6 ± 0.9	−0.3, 3.5	16.6; 0.96	6.8 ± 1.0	6.1 ± 1.8	0.7 ± 0.7	−0.9, 2.3	11.6; 0.50

Mean ± SD reported for all positions. TF = tight-five, LF = loose-forwards, IB = inside-backs, OB = outside-backs, Mean Diff (± SE) = mean difference ± standard error of mean difference, 95% CI = 95% confidence interval (lower limit, upper limit), % Diff = percentage difference, ES = effect size, Sum8SF = sum of eight-site skinfolds. * Statistically significant difference (*p* < 0.05) compared to LF.
